# Altered muscle-immune-gut axis response to eccentric exercise in men with well-controlled type 1 diabetes: a pilot study

**DOI:** 10.3389/fphys.2026.1855840

**Published:** 2026-07-22

**Authors:** Bartłomiej Matejko, Małgorzata Morawska-Tota, Sandra Mrozińska, Tomasz Klupa, Łukasz Tota

**Affiliations:** 1 Department of Metabolic Diseases, Jagiellonian University Medical College, Kraków, Poland; 2 University Hospital in Kraków, Kraków, Poland; 3 Department of Sports Medicine and Human Nutrition, University of Physical Culture, Kraków, Poland; 4 Department of Pathophysiology, Jagiellonian University Medical College, Kraków, Poland; 5 Department of Physiology and Biochemistry, University of Physical Culture, Kraków, Poland

**Keywords:** cytokines, eccentric exercise, exercise-induced muscle damage, inflammation, type 1 diabetes, zonulin

## Abstract

**Background:**

Eccentric-dominant exercise is a key component of many sports. It is also associated with exercise-induced muscle damage and activation of inflammatory responses. Physical activity, including sports, is one of the cornerstones in the treatment of type 1 diabetes (T1DM). However, the integrated response of the muscle–immune–gut axis to eccentric-dominant exercise in individuals with type 1 diabetes (T1D) remains poorly understood. This is the first study to evaluate the integrated muscle–immune–gut response to eccentric exercise in people with type 1 diabetes.

**Methods:**

The study comprised 39 physically active men (20–39 years old); 19 participants with well-controlled T1D, treated using continuous subcutaneous insulin infusion (CSII), and 20 healthy volunteers—control group. They performed a 30-minute downhill run (-10% inclination angle) at 60% VO_2_max. Blood samples were analyzed at baseline, 1-hour and 24-hours post-exercise assessed markers of muscle damage (LDH, myoglobin), inflammation (IL-6, TNF-α), hormonal responses (testosterone, cortisol), and gut-related response (zonulin).

**Results:**

However, in men with T1D, eccentric exercise triggers significantly more severe and prolonged inflammatory response, higher IL-6 concentrations, TNF-α and values of muscle damage markers, lasting up to 24 hours, compared to healthy controls. Increased cortisol levels may indicate a prolonged physiological response to exercise stress, while heightened zonulin levels may reflect changes in the gut-immune response following exercise with a predominance of eccentric contractions.

**Conclusion:**

In active men with T1D, eccentric exercise triggers more intense, prolonged inflammatory and metabolic responses compared to healthy controls. This underscores the muscle–immune–gut axis, necessitating individualized training loads and specific recovery strategies to optimize exercise adaptation in this population.

## 1 Introduction

Eccentric exercise is a key component of many sports, including trail running, team sports and activities requiring frequent changes in direction and braking. Compared to concentric contractions, it generates greater mechanical stress at a relatively lower metabolic cost, leading to microdamage of muscle fibers and triggering complex inflammatory and regenerative responses (Hyldahl and Hubal, 2014; Peake et al., 2017). This phenomenon—termed exercise-induced muscle damage (EIMD)—is characterized by increased levels of circulating markers such as LDH and myoglobin, as well as delayed activation of the immune axis within 24–48 hours following exercise (Paulsen et al., 2012; Peake et al., 2017).

This response is strictly regulated by proinflammatory cytokines, particularly interleukin 6 (IL-6) and tumor necrosis factor α (TNF-α). IL-6—a myokine secreted by contracting muscles—plays a metabolic role during exercise and also participates in the initiation and modulation of inflammatory as well as regenerative processes (Pedersen and Febbraio, 2008; Paulsen et al., 2012). TNF-α can enhance proteolysis and influence the balance between muscle catabolism and regeneration (Paulsen et al., 2012). In healthy individuals, this response is transient and adaptive. However, it remains unclear whether a similar balance is maintained in populations affected by chronic metabolic disorders.

Type 1 diabetes (T1D) is an autoimmune disease, the prevalence of which is increasing globally. Nonetheless, a growing number of individuals with T1D are beginning to engage in regular physical activity, including competitive training (Riddell et al., 2017). Physical activity is a cornerstone in the management of this disease due to its beneficial influence on metabolic control and cardiovascular health (Colberg et al., 2016; Riddell et al., 2017; Riddell et al., 2020). At the same time, T1D is associated with chronic low-grade inflammation, increased oxidative stress and skeletal muscle dysfunction—collectively referred to as diabetic myopathy (D’Souza et al., 2013; Riddell et al., 2017).

To date, research on T1D has been primarily focused on glycemic responses to aerobic exercise and strategies for modifying insulin therapy (Yardley et al., 2013; Riddell et al., 2017; Riddell et al., 2020), whereas the inflammatory response and markers of muscle damage following controlled eccentric loading remain poorly understood. Furthermore, gut–muscle axis has recently received increasing attention, as intense exercise can transiently increase intestinal barrier permeability due to splanchnic hypoperfusion (van Wijck et al., 2011; Costa et al., 2017). Zonulin—a physiological regulator of intestinal tight junctions—is commonly considered a marker of intestinal barrier integrity modulation; however, concerns have recently been raised regarding the reliability and specificity of commercially available assays used for its measurement (Fasano, 2011). Impaired intestinal barrier function and alterations in immune responses have also been reported in individuals with T1D (Fasano, 2011), suggesting a potentially enhanced intestinal response to exercise in this population.

The use of a standardized negative inclination treadmill running model allows for the isolation of EIMD’s mechanical component, while controlling exercise intensity relative to VO_2_max and enabling continuous monitoring of blood glucose levels (Hyldahl and Hubal, 2014; Matejko et al., 2021).

Understanding the integrated response of the muscle–immune–gut axis may be crucial for optimizing training recommendations and recovery strategies for individuals with type 1 diabetes. To our knowledge, there are no existing studies in which the integrated muscle–immune–gut response to eccentric exercise has been investigated in individuals with type 1 diabetes.

Therefore, the aim of this study was to evaluate integrated muscular, inflammatory, hormonal and gastrointestinal responses to eccentric-dominant exercise in physically active, well-controlled men with T1D using continuous subcutaneous insulin infusion (CSII), compared to a control group comprising healthy individuals. It was hypothesized that individuals with T1D would demonstrate a more pronounced and prolonged increase in the IL-6/TNF-α axis, markers of muscle damage and zonulin concentrations following eccentric exercise.

## 2 Methods

### 2.1 Study design and participants

**Figure 1:**
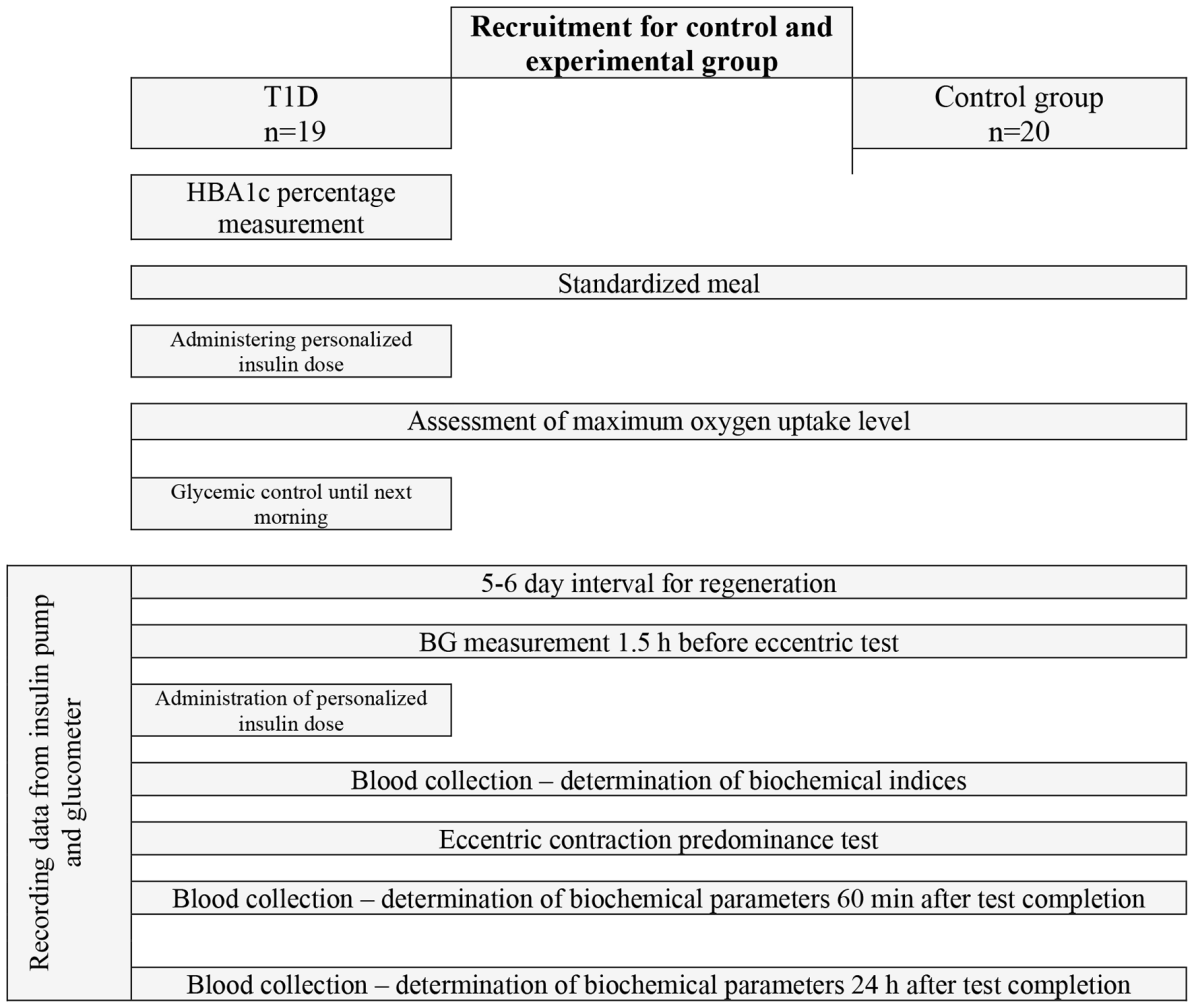
General scheme of research organization. BG, blood glucose; T1D , type 1 diabetes mellitus.

This is a prospective, controlled study, incorporating somatic, physiological as well as biochemical assessment. The study was designed as an extension of previously published data (Matejko et al., 2021). Male participants who consented to participate in the eccentric exercise protocol were fully informed of the study objectives and procedures and signed a written informed consent form. Exercise testing was performed under the constant supervision of a sports physiologist and an internal medicine physician. A general outline of the study organization is presented in **Figure 1**.

The study comprised 39 men aged 20–39 years: 19 with type 1 diabetes (T1D) treated with continuous subcutaneous insulin infusion (CSII), and 20 healthy volunteers serving as controls.

### 2.2 Inclusion and exclusion criteria

The inclusion criteria for both groups were as follows: age 18–40 years, performing regular physical activity (≥3 units per week for ≥12 months), no contraindications to moderate- to vigorous-intensity physical activity, no smoking or taking medications affecting metabolism and no ergogenic supplementation within six weeks prior to the study.

Additional inclusion criteria for the T1D group were: diagnosis of type 1 diabetes according to medical records, disease duration ≥2 years, CSII treatment for ≥6 months, stable metabolic control (HbA1c ≤8.0% during the last three months), no severe hypoglycemic episodes within the three months prior to testing, no advanced diabetic complications (proliferative retinopathy, autonomic neuropathy, clinically significant nephropathy), and no hospitalization due to ketoacidosis in the six months preceding the study.

Exclusion criteria for both groups included cardiovascular, respiratory or neurological diseases, acute infections within four weeks before testing, musculoskeletal injuries in the three months preceding the intervention, the use of nonsteroidal anti-inflammatory drugs within two weeks prior to testing and BMI >30 kg·m^-2^.

### 2.3 Experimental protocol

Participants were instructed to discontinue the use of supplements that could affect performance or biochemical parameters for six weeks prior to study initiation.

All patients except one used manual (non-automated) insulin pumps: 5 Accu-Chek Combo, 1 Accu-Chek Spirit, 6 Medtronic 722, 4 Medtronic 715, 2 Medtronic 754, and 1 Medtronic 640G, the only pump integrated with a CGM system that is partially automated and adjusts insulin delivery when hypoglycemia is imminent.

Ten days before the beginning of the physical capacity procedure, men in the T1D group used the Dexcom G4 continuous glucose monitoring system (display turned off), which was employed in this study to determine baseline characteristics.

HbA1c was determined using high-performance liquid chromatography (HPLC) in a hospital laboratory. All procedures were performed according to a strictly standardized protocol to ensure reproducible results.

### 2.4 Assessment of somatic indices

Body height (SECA 213; to the nearest 1 mm), body mass (Tanita BIA 545), and body composition were measured using bioelectrical impedance (AKERN BIA 101; CE0051). Percentage of body fat (F%), fat mass (FM) and lean body mass (LBM) were also evaluated. Body mass index (BMI) was calculated as the ratio of body mass in kilograms relative to body height in meters squared (kg/m²) (World Health Organization, 2000).

### 2.5 Assessment of aerobic capacity level

One week before the eccentric test, a graded test was conducted until refusal to directly determine peak oxygen uptake (VO_2_peak) and respiratory compensation point (RCP).

The test was performed on a mechanical treadmill (h/p/Cosmos Saturn 250/100R) in controlled environmental conditions (21-22 °C; humidity 40 ± 3%). The test began with a four-minute warm-up (7 km·h^-1^, 1% inclination), followed by an increase in treadmill speed by 1.0 km·h^-1^ every two minutes. The test continued until refusal of the subject to continue running. Respiratory gas analysis was performed using the Cortex MetaLyzer R3 ergospirometer. VO_2_, VCO_2_, VE and RER were recorded. VO_2_peak was defined as the highest recorded value. RCP was identified based on a non-linear increase in VE/VCO_2_ (Beaver et al., 1986). Heart rate was monitored telemetrically (Polar S610i). The detailed procedure of the physical fitness test is described in another publication (Matejko et al., 2021).

### 2.6 Safety of eccentric test procedure

All exercise tests were performed under the supervision of medical personnel. The eccentric test was scheduled for the week following the physical fitness test. Ninety minutes before the eccentric-dominant exercise test, if blood glucose levels were within the range of 70–250 mg/dL, basal insulin was reduced by 50-75% for two hours. Blood glucose was then measured immediately before and after the test using a Contour Plus glucometer.

### 2.7 Eccentric contraction predominance test

The eccentric test was performed on the H/P/Cosmos^®^ Saturn treadmill at a -10% inclination angle. The intensity was individually adjusted to 60% of VO_2_max (Ahmadi et al., 2008). The exercise duration was 30 minutes (± 30 s). To increase the mechanical component, participants performed the test with an additional load equal to 5% of their body mass (a backpack with weights selected to the nearest 0.1 kg). Heart rate was monitored telemetrically (Polar 610S). Continuous medical supervision was ensured during the test.

### 2.8 Assessment of biochemical indices

Venous blood was collected from the basilic vein at three time points: immediately before the exercise test comprising predominantly eccentric contractions, one hour after its completion, and 24 hours after the exercise. The first blood samples were collected in the morning—after at least a 10-hour overnight fast—to limit the influence of circadian rhythms and dietary factors on the measured parameters, while the remaining two collections were conducted in the afternoon.

Blood was collected into EDTA-containing tubes and centrifuged at 2,000 rpm for 10 minutes at 4 °C. After centrifugation, plasma was separated and stored at -80 °C until analysis.

Serum concentrations of lactate dehydrogenase (LDH), myoglobin, interleukin-6 (IL-6), tumor necrosis factor alpha (TNF-α), total testosterone, cortisol, and zonulin were determined using commercially available enzyme-linked immunosorbent assay (ELISA) kits (Cloud-Clone Corp., Wuhan, China) according to the manufacturer’s instructions.

Assay sensitivity and intra- and inter-assay coefficients of variation were within the manufacturer’s specifications (<10%). All analyses were performed by the same person, blinded to group assignment and using the same reagent lots to minimize analytical variability.

All procedures were carried out in accordance with the 1964 Declaration of Helsinki, and its later amendments. The project received approval from the local bioethics committee at the District Medical Chamber (approval No. 1072.6120.113.2017).

### 2.9 Statistical analysis

All available data were used in computing means and SDs. No imputation method was applied for missing data. For comparisons of two independent groups, the Student’s or Welch’s *t*-tests were used for normally distributed continuous variables (assessed via the Shapiro-Wilk test); otherwise, the Mann-Whitney U test was applied. For comparisons of two dependent groups, the paired *t*-test or Wilcoxon signed-rank test were used, as appropriate. All P values are two tailed. Analyses were performed with R (version 4.5.1) and RStudio (version 1.3.959). The level of statistical significance was adopted at *p* < 0.05.

## 3 Results

Basic somatic parameters of the study participants are presented in **Table 1**. The mean age of the study participants was 25.3 ± 5.4 years in the group of men with type 1 diabetes and 24.3 ± 2.5 years in the control group. There were no significant differences between the groups in body height or mass, body mass index (BMI), lean body mass (LBM), fat mass (FM) or body fat percentage (F%) (*p*>0.05). Baseline characteristics of metabolic control in participants with type 1 diabetes are presented in **Table 2**.

**Table 1:** Somatic indices of studied groups.

Subjects indices	T1D	Control group	*p-value*
Age [years]	25.3±5.4	24.3±2.5	*0.910*
LBM [kg]	64.9±6.2	63.1±6.0	*0.360*
FM [kg]	13.9±5.4	10.6±3.8	*0.074*
F% [%]	17.3±4.9	14.3±4.2	*0.140*
BMI [kg/m^2^]	23.9±2.3	22.8±2.0	*0.148*

BH, body height; BM, body mass; BMI, body mass index; FM, fat mass; F%, fat percentage; LBM, lean body mass; T1D, type 1 diabetes mellitus, *p*<0.05.

**Table 2:** Clinical characteristics and selected parameters of continuous glucose monitoring in T1D patients.

Variable				
Statistic	Diabetes duration [years]	Time of CSII [years]	HbA1c [%]	Daily Insulin dose [IU]
Mean ± SD	12.2 ± 6.0	7.3 ± 4.3	6.9 ± 0.7	54.9 ± 13.6
	DDI/kg [IU/kg]	Median glycaemia from CGM [mg/dL]	Mean Time in Range [mg/dL]	Mean time Above 180 mg/dl
Mean ± SD	0.7 ± 0.1	137.9 ± 20.5	63.1 ± 8.3	27.1 ± 11.1
	Time above >250 mg/dl	Time spent below 54 mg/dL	Time spent below 70 mg/dL	Time spent below 54 mg/dL
Mean ± SD	8.8±6.4	3.5 ± 3.1	9.8 ± 7.1	3.5 ± 3.1
	Number of steps per day [N]			
Mean ± SD	8771.9 ± 2695			

CV, coefficient of variation; CGM, continuous glucose monitoring; CSII, continuous subcutaneous insulin infusion; DDI/kg, daily dose of insulin per kg of body mass.

Mean BG values through the eccentric test were as follows: 1.5 h before the exercise 196.2 ± 44.6 mg/dl just before the test 198.5 ± 50.5 mg/dl and after the test 134.6 ± 44.4 mg/dl. No serious adverse events or incidents requiring medical intervention were recorded during the study procedures.

The participants were characterized by optimal HbA1c levels but spent more time in hypoglycemia than recommended. Physical fitness level—assessed based on step counts—could be considered moderate (**Table 2**).

Overall, men with type 1 diabetes exhibited lower exercise capacity compared to the healthy controls. The majority of parameters measured at both the respiratory compensation point (RCP) and maximal effort were significantly reduced in the T1D group, indicating diminished physiological responses to exercise (**Table 3**).

**Table 3:** Physiological indices obtained from physical capacity exercise test.

Indices	T1D group		Control group		*p*	
	Max	RCP	Max	RCP	Max	RCP
t [min]	16.8±2.7	8.6±1.9	18.5±3.1	11.6±2.8	0.078	<0.001
v [km · h^–1^]	12.4±1.3	8.3±1.0	16.2±2.2	12.4±1.9	<0.001	<0.001
HR [beats · min^–1^]	197.9±7.1	167.2±6.7	191.4±9.6	169.8±8.9	0.005	0.322
VO_2_peak [L · min^–1^]	3.6±0.4	2.6±0.4	4.2±0.5	3.4±0.4	<0.001	<0.001
VO_2_peak [mL · kg^–1^ · min^–1^]	45.4±4.4	32.6±4.7	56.7±7.6	45.9±6.1	<0.001	<0.001
Ve [L · min^–1^]	132.1±20.6	69.0±11.1	153.5±21.6	93.9±15.7	<0.001	<0.001

HR, heart rate; Max, maximal index level; T1D, type 1 diabetes mellitus; RCP, respiratory compensation point; *t*, working time in graded treadmill test; v, speed in the graded treadmill test; Ve, respiratory ventilation per minute VO_2_peak, maximal oxygen uptake: global [L · min^–1^], [mL · kg^–1^ · min^–1.^

Changes occurring in biochemical parameters with regard to inflammatory response, muscle damage, hormonal response and markers regarding the intestinal response to exercise with a predominance of eccentric contractions in both study groups are presented in **Table 4**. The obtained results indicate reduced physical performance in people with T1D and increased changes in the inflammatory, muscle and intestinal response following eccentric exercise in comparison to the control group.

**Table 4:** Biochemical indices in studied groups (T1D vs. control) at individual time points.

Biochemical parameter	T1DM		Healthy males			*P*-value (between group)			*P*-value (between group)			*P*-value (between group)	
	Baseline	60 min		24h	Baseline		60 min	24h		Baseline	60 min		24h
IL-6 [pg·mL^-1^]	7.3 ± 1.8	26.4 ± 9.0		68.4 ± 15.5	3.6 ± 1.6		7.6 ± 2.5	36.3 [32.6-56.1]		<0.001	<0.001		<0.001
Cortisol [ng·mL^-1^]	154.8 ± 36.8	283.9 ± 56.3		189.7 ± 51.6	145.7 ± 37.0		228.0 ± 57.1	150.0 ± 38.0		0.446	0.004		0.008
LDH [U·L^-1^]	198.9 ± 54.0	298.5 ± 46.4		230.8 ± 73.9	152.4 ± 41.1		206.0 ± 43.4	151.0 ± 27.4		0.004	<0.001		<0.001
Mb [ng·L^-1^]	25.4 ± 11.1	165.3 [143.4-254.5]		84.4 ± 27.7	26.3 ± 10.9		70.9 [58.1-91.8]	27.7 ± 9.1		0.789	<0.001		<0.001
Testosterone [ng·ml^-1^]	4.7 ± 1.0	4.1 ± 0.8		4.7 ± 1.0	4.2 ± 0.8		4.0 ± 1.0	4.6 ± 0.9		0.116	0.560		0.688
TNF [pg·mL^-1^]	11.4 ± 3.1	16.8 ± 3.2		11.1 ± 2.2	4.9 ±1.1		6.2 ±1.6	4.8 ± 1.2		<0.001	<0.001		<0.001
Zonulin [ng·mL^-1^]	20.9 ± 7.3	36.1 ± 9.5		40.4 ± 9.7	18.3 ± 7.3		27.5 ± 9.7	20.5 ± 8.5		0.361	0.008		<0.001

IL-6, interleukin-6; LDH, lactate dehydrogenase; Mb, myoglobin; TNF-α, tumor necrosis factor alpha.

## 4 Discussion

The aim of this study was to evaluate the integrated muscle, inflammatory, hormonal and gut responses to eccentric-dominant exercise (e.g. trail running, team sports requiring frequent changes of direction and strength training with emphasis on the eccentric phase) in physically active, well-controlled men with T1D treated with continuous subcutaneous insulin infusion (CSII), compared to a healthy control group. It was hypothesized that T1D would be associated with a more pronounced and prolonged muscle–immune–gut response, as manifested by greater activation of the axis, higher values of muscle damage markers and increased zonulin concentrations following eccentric exercise.

The primary finding of this study is that physically active, well-controlled men with T1D (mean HbA1c <7%) demonstrate a similar overall pattern and time course of response to standardized eccentric exercise compared to healthy controls, as reflected by consistent directionality in markers of inflammation, hormones and muscle damage. However, this response was more severe and prolonged in the T1D group (Colberg et al., 2016; Riddell et al., 2017).

It should be noted that the study population comprised physically active men with well-controlled type 1 diabetes, as indicated by favorable mean CGM metrics (**Table 2**). This profile is not typical of the general T1D population and should be considered when interpreting the findings (Riddell et al., 2020). The most pronounced difference between the groups was a stronger and more prolonged inflammatory response in subjects with T1D, expressed by higher concentrations of IL-6 and TNF-α after eccentric exercise (**Table 3**). IL-6—a myokine produced by contracting muscle fibers—plays an important role in the regulation of energy substrate metabolism during exercise, but also participates in the initiation and modulation of the inflammatory response associated with EIMD (Pedersen and Febbraio, 2008; Paulsen et al., 2012; Peake et al., 2017). TNF-α is a key inflammatory mediator that can enhance proteolysis and influence muscle catabolism (Paulsen et al., 2012). One should note that T1D is also characterised by a higher-than-recommended time in the hypoglycemia range, which may exacerbate exercise-induced stress and contribute to the amplified inflammatory response observed in this group (Kiec-Wilk et al., 2016).

Despite standardizing exercise intensity relative to individual VO_2_max, participants with lower fitness levels may experience higher relative physiological and metabolic stress. A significant methodological aspect is the method applied for determining exercise intensity. Although the load during the eccentric test has been standardized to individual VO_2_max, contemporary research indicates that physiological exercise thresholds, such as respiratory compensation point (RCP), may more accurately reflect the actual exercise intensity domain than values ​​expressed as a percentage of VO_2_max (Iannetta et al., 2020). This increased load often exacerbates post-exercise responses, particularly inflammation, which is closely linked to metabolic demand and training status (Fischer, 2006). However, an additional analysis performed in the present study did not indicate a greater relative physiological strain in participants with T1D when exercise intensity was expressed relative to individual RCP values.

Lower aerobic capacity observed in the T1D group is associated with reduced efficiency of oxidative metabolism and a greater contribution of anaerobic metabolism, which may lead to greater accumulation of metabolic products and increased oxidative stress. In the context of eccentric exercise, this may result in greater susceptibility to muscle fiber damage and a more pronounced inflammatory response, as described in the literature on exercise-induced muscle damage and cytokine activation (Pedersen and Febbraio, 2008; Paulsen et al., 2012; Peake et al., 2017). Furthermore, training level has been shown to modulate immune response to exercise, with higher aerobic capacity associated with a more controlled and shorter inflammatory response (Fischer, 2006; Gleeson, 2007).

It is also worth emphasizing that participants with T1D demonstrated a moderate level of daily physical activity, expressed by a mean step number of over 8,500 per day. This suggests that the observed differences in aerobic capacity and post-exercise response cannot be fully explained by lower levels of habitual activity, but most likely result from an interaction between training level and metabolic disturbances characteristic of T1D (Fischer, 2006; Gleeson, 2007; Giacco and Brownlee, 2010; D’Souza et al., 2013).

In the T1D population, chronic low-grade inflammation and elevated levels of proinflammatory cytokines are observed even at rest (Pedersen and Febbraio, 2008; Riddell et al., 2017). Therefore, it may be assumed that eccentric exercise in this group acts as a stimulus superimposed on the already existing immune activation, leading to an amplification of the IL-6/TNF-α axis response. This phenomenon may be important for regenerative processes, as excessive or prolonged activation of this axis can disturb the balance between muscle fiber degeneration and regeneration (Paulsen et al., 2012).

The increases in LDH and myoglobin after exercise confirm the occurrence of EIMD in accordance with the profile of response to eccentric loads described in the literature (Paulsen et al., 2012; Hyldahl and Hubal, 2014; Peake et al., 2017). Higher post-exercise values and slower normalization in the T1D group may indicate greater susceptibility of muscle tissue to mechanical stress or less effective regenerative mechanisms (Ahmadi et al., 2008). The observed statistically significant differences between groups (before: *p* = 0.004; 60 min after: *p* < 0.001; 24 h after: *p* < 0.001) suggest that T1D may modify both the severity and duration of the muscle response to eccentric loads.

The phenomenon of diabetic myopathy—which includes satellite cell dysfunction, reduced regenerative capacity and structural changes in skeletal muscles in diabetes—has been described in the literature (D’Souza et al., 2013). Combined with increased inflammation, this may explain the prolonged response of muscle damage markers observed in our study. In sports practice, this potentially indicates the need for extended recovery periods among individuals with T1D following intense eccentric exercise (Peake et al., 2017).

Activation of the hypothalamic–pituitary–adrenal (HPA) axis was evident in both groups as increased cortisol after exercise, representing a physiological response to exercise stress. However, the persistence of elevated cortisol levels after 24 hours in the T1D group may be indicative of slower recovery from the stress response and potentially increased allostatic load (McEwen, 2006). Nonetheless, it should be noted that no episodes of hypoglycemia or ketone bodies requiring medical intervention were observed or reported during the 24-hour period following the test. This suggests that the noted changes in cortisol concentration were primarily related to exercise response, although the influence of unrecorded changes in glycaemia cannot be completely dismissed.

Although CSII therapy allows for flexible adjustment of insulin delivery to exercise (Matejko et al., 2021), it does not fully replicate the physiological dynamics of insulin secretion (Sherr and Tamborlane, 2016). As an anabolic hormone, insulin influences protein metabolism and regenerative processes (Riddell et al., 2017), and differences in its physiological profile may indirectly modulate the catabolic response post-exercise. The observed transient decrease in testosterone may indicate a short-term shift towards a catabolic environment, which—combined with a prolonged cortisol response—can affect recovery rate (Sherr and Tamborlane, 2016).

One of the most significant and novel findings of this study was the greater and more sustained increase in zonulin after eccentric exercise in the T1D group. Intense exercise can lead to transient splanchnic hypoperfusion and intestinal hypoxia, resulting in alterations of intestinal barrier function and gut epithelial integrity (van Wijck et al., 2011; Costa et al., 2017). Zonulin—as a regulator of tight junctions—plays a key role in modulating the integrity of the intestinal epithelium (Fasano, 2011). However, it should be underlined that only total testosterone concentration was measured in this study. The lack of sex hormone-binding globulin (SHBG) levels prevents the assessment of free or bioavailable testosterone, therefore, the obtained results should be interpreted solely in relation to changes in total testosterone concentration (Tomar et al., 2003).

Furthermore, it should be emphasized that the interpretation of circulating zonulin concentration as a direct marker of intestinal barrier permeability remains a matter of debate (Scheffler et al., 2018; Massier et al., 2021). In recent years, it has been shown that widely used commercial ELISA kits can detect not only pre-haptoglobin-2 (zonulin), but also other proteins structurally related to the zonulin-like protein family. This limits the specificity of the biomarker as an indicator of intestinal barrier integrity. Therefore, the increase in zonulin concentration observed in the current study should be interpreted with caution and considered as an indicator of changes related to the gut-immune axis response or exercise-induced epithelial stress rather than as unequivocal evidence of intestinal barrier dysfunction. An additional limitation of the study is the lack of determination of other intestinal permeability markers, such as intestinal fatty acid-binding protein (I-FABP), lipopolysaccharide (LPS) or lipopolysaccharide binding protein (LBP) commonly used to assess intestinal barrier integrity, enterocyte damage and endotoxin translocation. They could also further provide more direct information on intestinal barrier function (Scheffler et al., 2018; Ajamian et al., 2019).

Changes in the regulation of intestinal barrier function and immune response have been previously described in individuals with type 1 diabetes (Pedersen and Febbraio, 2008; Fasano, 2011; Riddell et al., 2017). Chronic low-grade inflammation and elevated levels of proinflammatory cytokines (Pedersen and Febbraio, 2008; Riddell et al., 2017) may influence the expression of tight junction proteins and promote their destabilization. Eccentric-dominant exercise, through redistribution of blood flow and induction of oxidative stress (van Wijck et al., 2011; Costa et al., 2017), potentially acts as a factor revealing the existing vulnerability of the gut barrier in T1D.

The observed increase in zonulin concentration may suggest altered gut-immune responses potentially contributing to immune activation. However, this interpretation should be made with caution, given the limitations of circulating zonulin as an indirect marker of intestinal barrier permeability (Scheffler et al., 2018; Ajamian et al., 2019).

Physical activity remains a cornerstone of T1D therapy (Colberg et al., 2016; Riddell et al., 2017), and an increasing number of individuals with this condition participate in competitive and recreational sports (Riddell et al., 2020).

Further research is required to determine whether repeated episodes of increased inflammatory and gastrointestinal responses influence long-term training adaptations and the risk of overload in individuals with T1D, particularly those with poorer glycemic control.

### 4.1 Study limitations

This study has several limitations that should be considered when interpreting the results.

First of all, the relatively small sample size and the homogeneous nature of the study group limit generalizability of the results. Participants with T1D were similar in terms of treatment modality (CSII), metabolic control and age, making it difficult to extrapolate the results to a more diverse population of people with type 1 diabetes. Secondly, differences in aerobic capacity between participants may have influenced the size of the physiological and inflammatory response, despite standardizing exercise intensity to individual VO_2_max. Furthermore, participants with T1D had a slightly higher frequency of hypoglycemic episodes than recommended in current clinical guidelines, which may have influenced metabolic stability and the observed post-exercise responses. Additionally, daily physical activity levels were assessed only in the T1D group, and the lack of comparable data for the control group limits the ability to fully interpret the role of habitual activity in shaping the observed differences. Finally, the study was conducted exclusively among men. This approach aimed to ensure greater homogeneity in the study population and to limit the influence of sex-related differences, particularly those related to hormonal response, inflammatory processes and muscle response to exercise. Therefore, the obtained results cannot be directly generalized to the female population. Another limitation of the study is the use of exercise intensity defined as a percentage of VO_2_max rather than in relation to individual respiratory compensation point (RCP). Although additional analysis did not reveal a greater relative physiological burden in the T1D group, future studies should consider using RCP for more precise standardization of exercise intensity.

An additional limitation of the present study is the interpretation of circulating zonulin concentrations. Current data indicate that commercially available ELISA kits may measure proteins related to the zonulin family, not exclusively pre-haptoglobin-2, which restricts the specificity of this marker as an indicator of intestinal barrier permeability. Furthermore, additional markers of intestinal barrier function—such as I-FABP, LPS or LBP—were not measured in the study. Therefore, the observed changes in zonulin concentrations should be interpreted with caution and considered as an indirect indicator of a response related to the gut-immune axis rather than unequivocal evidence of impaired intestinal barrier integrity.

Furthermore, the statistical approach used relied on multiple comparisons between time points and groups. Although this method allows for direct interpretation of changes occurring at individual measurement points, it does not fully account for the dependencies resulting from repeated measurements carried out on the same participants. Future studies involving larger groups of participants would benefit from the use of mixed models, which would allow for simultaneous assessment of the effects of time, group, and their interactions.

Additionally, no formal sample size calculation was performed before initiating the study because the pilot project was experimental in nature. Therefore, the results should be interpreted as preliminary and require confirmation in larger studies involving more diverse groups of individuals with type 1 diabetes.

Moreover, sex hormone-binding globulin (SHBG) levels were not measured in this study, which precludes the assessment of free and bioavailable testosterone levels. Therefore, the interpretation of hormonal response was limited to changes in total testosterone levels.

## 5 Conclusion

The results indicate that physically active, well-controlled men with type 1 diabetes respond to eccentric-dominant exercise according to a similar response pattern observed in healthy individuals, but this response is characterized by greater intensity and prolonged duration. This regards markers of muscle damage, inflammatory and hormonal responses, as well as biomarkers related to gut-immune axis response.

The observed differences suggest that individuals with T1D may experience increased activation of the muscle-immune-gut axis and slower post-exercise recovery, despite demonstrating similar levels of physical activity and good metabolic control.

These results permit underlining the significance of individualizing training loads and recovery strategies in this population and imply a potential role for the muscle-immune-gut axis in exercise adaptation in individuals with type 1 diabetes; however, further research in larger and more diverse cohorts of individuals with this condition is warranted to confirm the generalizability of these findings. The obtained results may constitute the basis for optimizing training recommendations for individuals with type 1 diabetes.

## Data Availability

The datasets presented in this study can be found in online repositories. The names of the repository/repositories and accession number(s) can be found in the article/**Supplementary Material**.
