# Supplementary material for: Altered muscle-immune-gut axis response to eccentric exercise in men with well-controlled type 1 diabetes: a pilot study
Source: Front Physiol. 2026 Jul 22;17:1855840. doi: 10.3389/fphys.2026.1855840 (PMC13437349; doi:10.3389/fphys.2026.1855840)
Supplement: Supplementary file 1 [file DataSheet1.pdf]

L.p.	HRmax (beats·min⁻¹)	VO2max [L·min-1]	VO2max/kg	v RCP [km/h]	HR RCP[sk/min]	VO2 RCP [L·min-1]	VO2/kg RCP	Mean VO_2 during the 30-min test (L·min⁻¹)	Mean HR during the 30-min exercise test (beats·min⁻¹)	Group
1	199	4,33	41,1597	8	158	3,08	29,2776	1,94	152	T1D
2	195	3,41	45,4667	7,5	166	2,23	29,7333	1,69	137	T1D
3	198	3,78	46,6667	9	172	2,87	35,4321	2,08	148	T1D
4	194	3,07	45,549	8	162	2,06	30,5638	1,64	149	T1D
5	204	3,86	51,8121	9	169	2,49	33,4228	2,4	170	T1D
6	208	3,56	44,4444	7	174	2,68	33,4582	2,44	174	T1D
7	184	4,01	51,0828	10	163	3,24	41,2739	2,43	160	T1D
8	195	3,41	45,4667	7,5	166	2,23	29,7333	2,3	171	T1D
9	193	3,89	48,0841	8,7	163	2,85	35,2287	2,83	160	T1D
10	200	3,1	37,6671	7,2	165	2,1	25,5164	2	164	T1D
11	204	2,95	44,0957	8	167	2,15	32,1375	2,04	166	T1D
12	204	2,62	39,697	7,5	167	1,83	27,7273	1,84	170	T1D
13	192	3,8	41,8502	7,8	160	2,39	26,3216	2,18	161	T1D
14	198	3,79	44,7991	7,8	171	2,6	30,7329	2,31	172	T1D
15	203	3,99	51,0883	9,8	176	3,01	38,5403	2,62	172	T1D
16	211	3,07	41,4865	8	186	2,32	31,3514	2,04	178	T1D
17	192	3,78	49,0272	8	159	2,82	36,5759	2,17	156	T1D
18	185	3,67	52,8818	10,8	164	2,97	42,7954	2,38	162	T1D
19	202	3,73	41,17	8	169	2,73	30,1325	2,31	161	T1D
20	179	3,84	48,1	10,7	162	3,09	38,7	2,81	155	
21	190	3,88	47,5	10,9	156	3,16	38,7	2,85	159	
22	191	4,24	54,6	15,6	180	3,72	47,9	3,67	176	
23	180	4,21	46,5	10,7	157	3,35	37	3,4	160	
24	202	4,35	60,9	12,3	180	3,24	45,4	3,5	177	
25	183	4,89	70,8	13,6	166	3,47	50,2	3,5	169	
26	191	5,04	61,9	13,4	171	4,13	50,7	4,2	165	
27	186	3,24	54,5	11,8	169	2,83	47,6	2,9	171	
28	193	4,16	63,6	11,8	160	3,3	50,5	3	155	
29	199	4,29	67,8	14,8	177	3,26	51,4	2,9	170	
30	189	4,57	60,6	14,8	172	3,95	53,8	4	173	
31	204	3,82	60,3	14	189	3,31	52,2	3,5	177	
32	185	4,44	63,4	14,5	166	3,66	52,3	3,9	170	
33	220	4,34	54,2	10,2	177	3,06	38,3	3	170	
34	191	3,28	48	10,5	171	2,81	41,2	2,85	168	
35	183	4,66	62,9	14,6	159	3,9	52,6	3,3	155	
36	193	3,81	48,8	11	163	2,91	37,2	2,7	160	
37	195	4,52	59	13,5	180	3,8	49,6	3,01	173	
38	182	3,47	44,5	9,1	171	3	38,4	2,8	168	
39	192	4,13	55,4	10,6	169	3,37	45,2	3,3	165	
